# Supplementary material for: Synergistic IL-6 and IL-8 paracrine signalling pathway infers a strategy to inhibit tumour cell migration
Source: Nat Commun. 2017 May 26;8:15584. doi: 10.1038/ncomms15584 (PMC5458548; doi:10.1038/ncomms15584)
Supplement: Supplementary Information — Supplementary Figures and Supplementary Tables [file ncomms15584-s1.pdf]

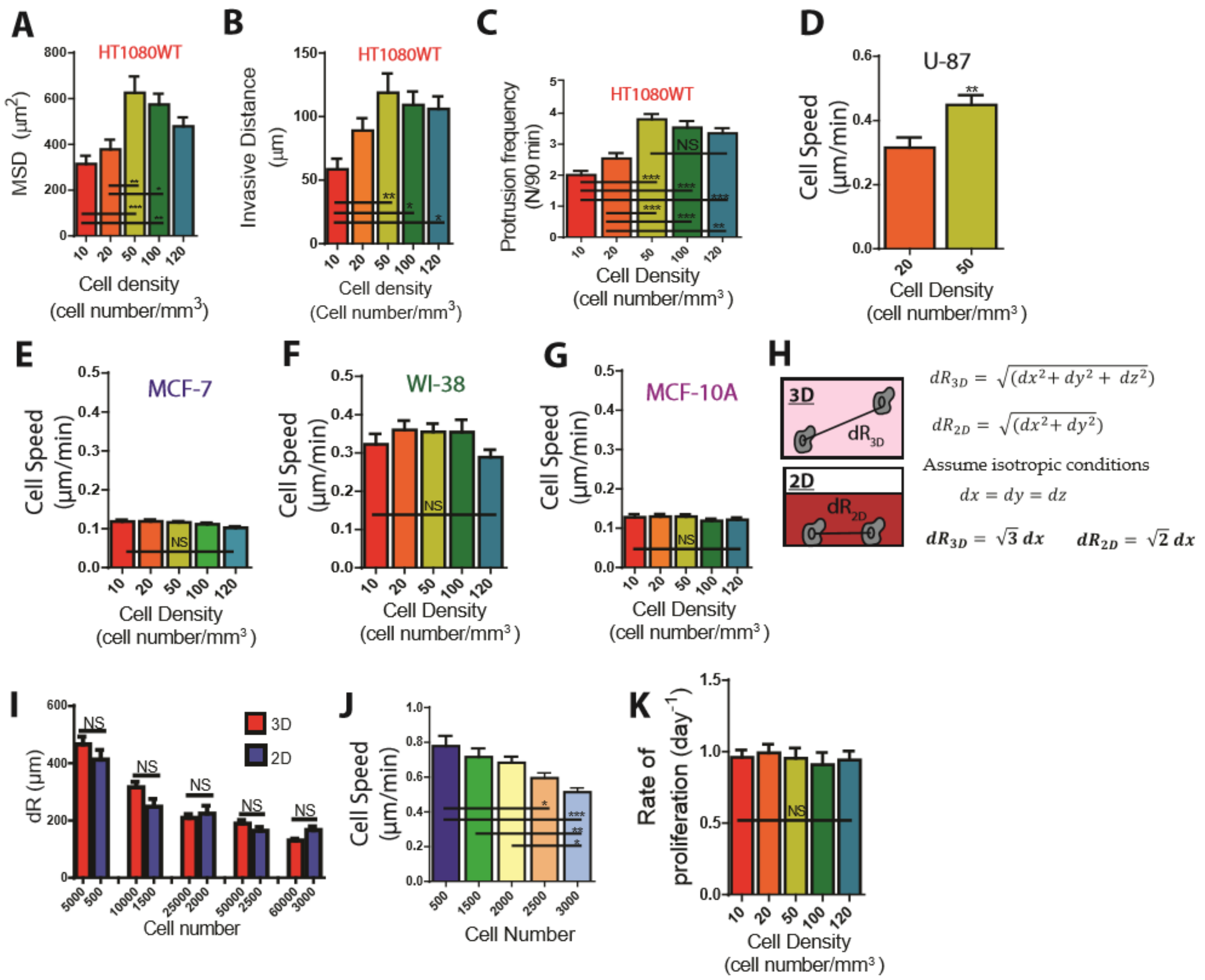

Supplementary Figure 1: Influence of cell density on migration. A,B. Migration parameters of fibrosarcoma cells measured at a time lag of 2 min at different seeding densities. **C.** Relationship between protrusion frequency and cell density: Protrusion frequency increases with cell density and plateaus at a threshold density of 50 cells/ mm^3. **E-G**. Cell speed measured at a time lag of 2 min at different seeding densities. **H** and **I**. Relating 3D cell densities to 2D cell densities. **J.** 2D cell speed measured at a time lag of 2 min at different cell numbers that relate to the 3D cell densities. **K.** Rate of proliferation remains unchanged with increasing cell density. In all panels, data was obtained from 3 independent experiments and is represented as mean ± s.e.m. *P<0.05; **P<0.01; ***P<0.001(ANOVA)

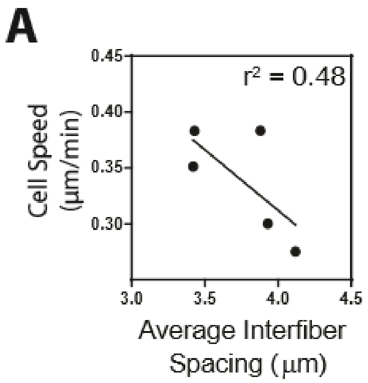

Supplementary Figure 2: Physical cues that influence cell speed A. Correlation plot of cell speed vs. average interfiber spacing. Data was obtained from 3 independent experiments and is represented as mean.

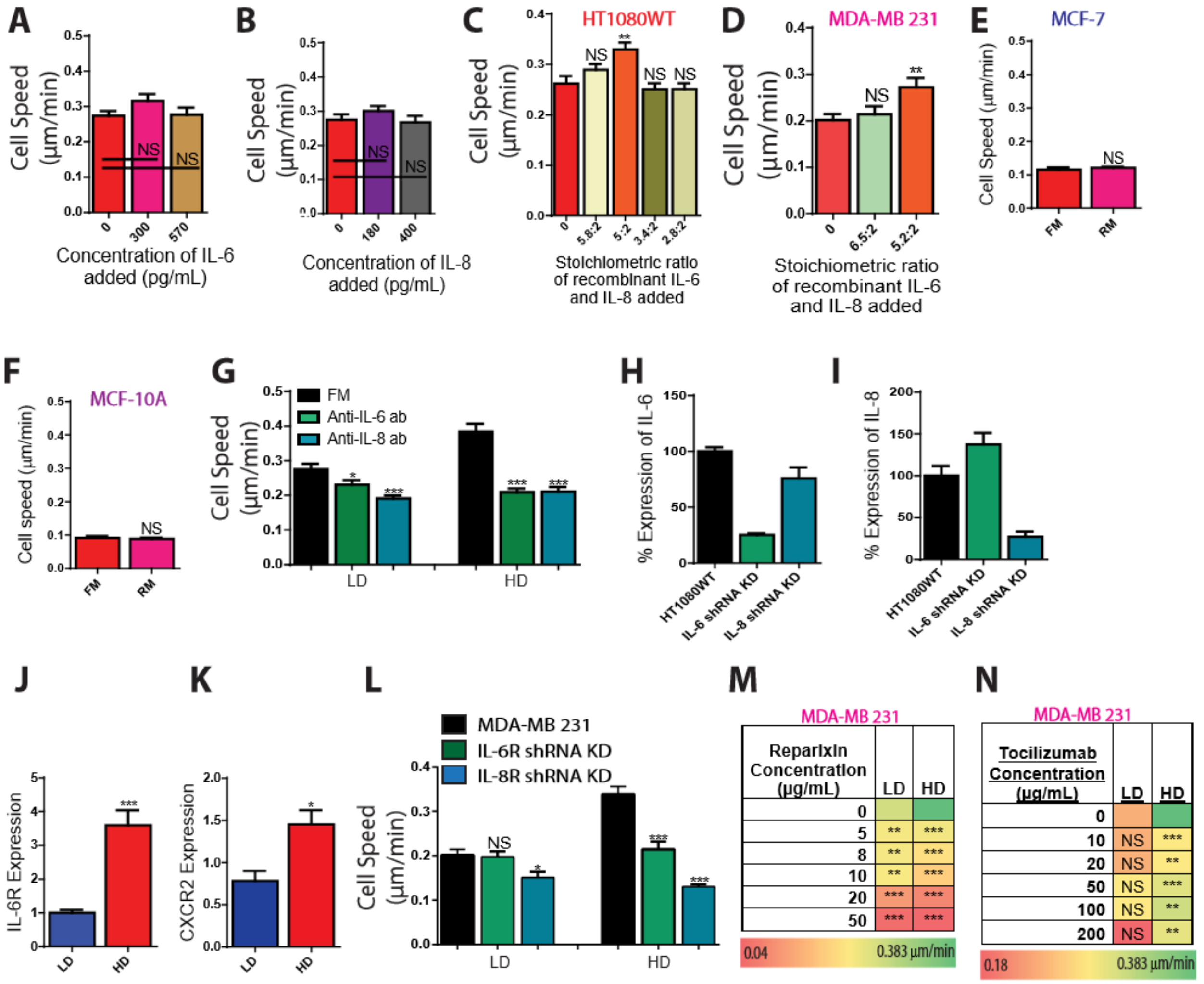

Supplementary Figure 3: Biochemical cues that regulate cell density-dependent migration. **A** and **B**. The addition of high concentrations of recombinant IL-6 alone or recombinant IL-8 alone do not increase cell speed. **C** and **D.** The addition of recombinant IL-6 and IL-8 in combination at ratios other than 5:2 does not induce the high speed observed at high densities. **E** and **F.** The addition of recombinant IL-6 and IL-8 in combination does not enhance migration in non-metastatic cells. **G.** Decreased velocity of cells exposed to specific IL-6 and IL-8 functional antibodies at LD (ρ =10) and HD (ρ=50) compared to untreated control cells. **H** and **I.** Decreased expression of IL-6 and IL-8 in shRNA mediated knockdowns. **J** and **K.** Expression of IL-6R and CXCR2 (IL-8R) as quantified by PCR. **L.** Decreased speed of the IL-6R and IL-8R knockdown cells at LD (ρ =10) and HD (ρ =50). **M** and **N**. Individually, Tocilizumab and Reparixin decreased cell speed of human carcinoma cells embedded in a 3D matrix at LD (ρ =10) and HD (ρ =50) compared to cells exposed to fresh medium (0). In all panels, data was

obtained from 3 independent experiments and is represented as mean ± s.e.m. *P<0.05; **P<0.01; ***P<0.001(ANOVA)

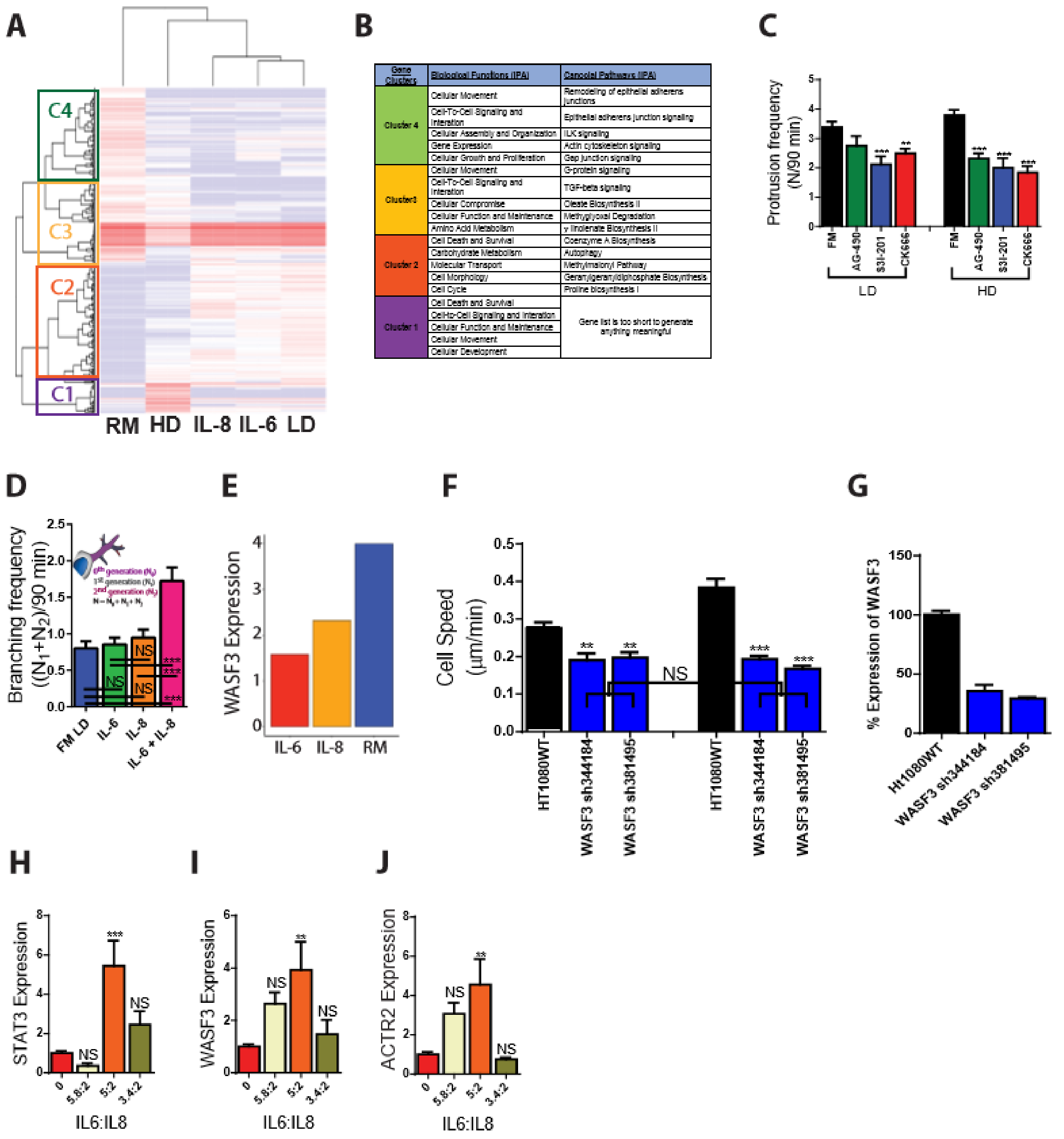

Supplementary Figure 4: Proposed mechanism. A. Heat map demonstrating the difference between gene ontology categories**. B.** Table demonstrating gene ontology categories**. C.** Decreased protrusion activity of human fibrosarcoma cells embedded in a 3D matrix exposed to JAK2 inhibitor, AG-490, STAT3 inhibitor, S3I-201, and Arp2/3 complex inhibitor, CK 666, at LD (ρ =10) and HD (ρ =50) compared to cells exposed to fresh medium (FM). **D.** The addition of recombinant IL-6 and IL-8 alone does not increase branching frequency, however the

addition of recombinant IL-6 and IL-8 in combination at the precise concentrations found in a matrix containing a high density of 50 cells/mm^3 (RM) significantly increases branching frequency. **E.** Increased expression of WASF3 in cells exposed to recombinant IL-6 and IL-8 in combination at the precise concentrations found in a matrix containing a high density of 50 cells/mm^3. **F.** Decreased velocity of cells depleted of WASF3 at LD (ρ =10) and HD (ρ=50) compared to wildtype cells. **G.** Decreased mRNA expression of WASF3 in shRNA mediated knockdowns in HT1080 fibrosacroma. **H-J.** Maximal expression of STAT3, JAK2, and ACTR2 were observed when recombinant IL-6 and IL-8 were added in combination at a ratio of 5:2. In panels C,D, F-J, data was obtained from 3 independent experiments and is represented as mean ± s.e.m. *P<0.05; **P<0.01; ***P<0.001(ANOVA). RNA Seq data (A,E) was obtained from a single experiment

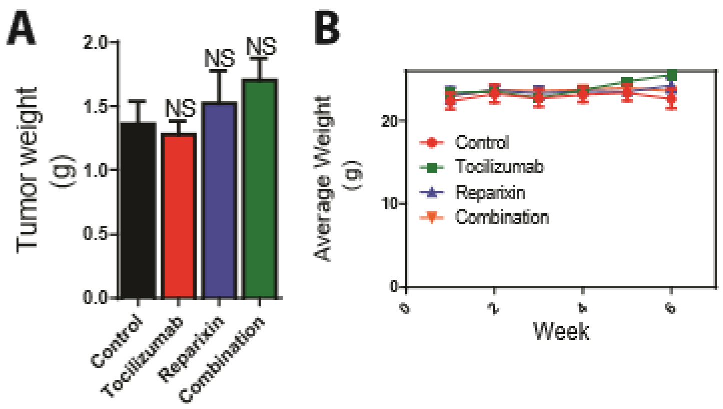

Supplementary Figure 5: Validation in mouse xenograft model. A. final tumor weight measured in grams. **B.** Average weight of mice measured in grams. In all panels, data was obtained from 5 individual mice in each set and is represented as mean ± s.e.m. *P<0.05; **P<0.01; ***P<0.001(ANOVA)

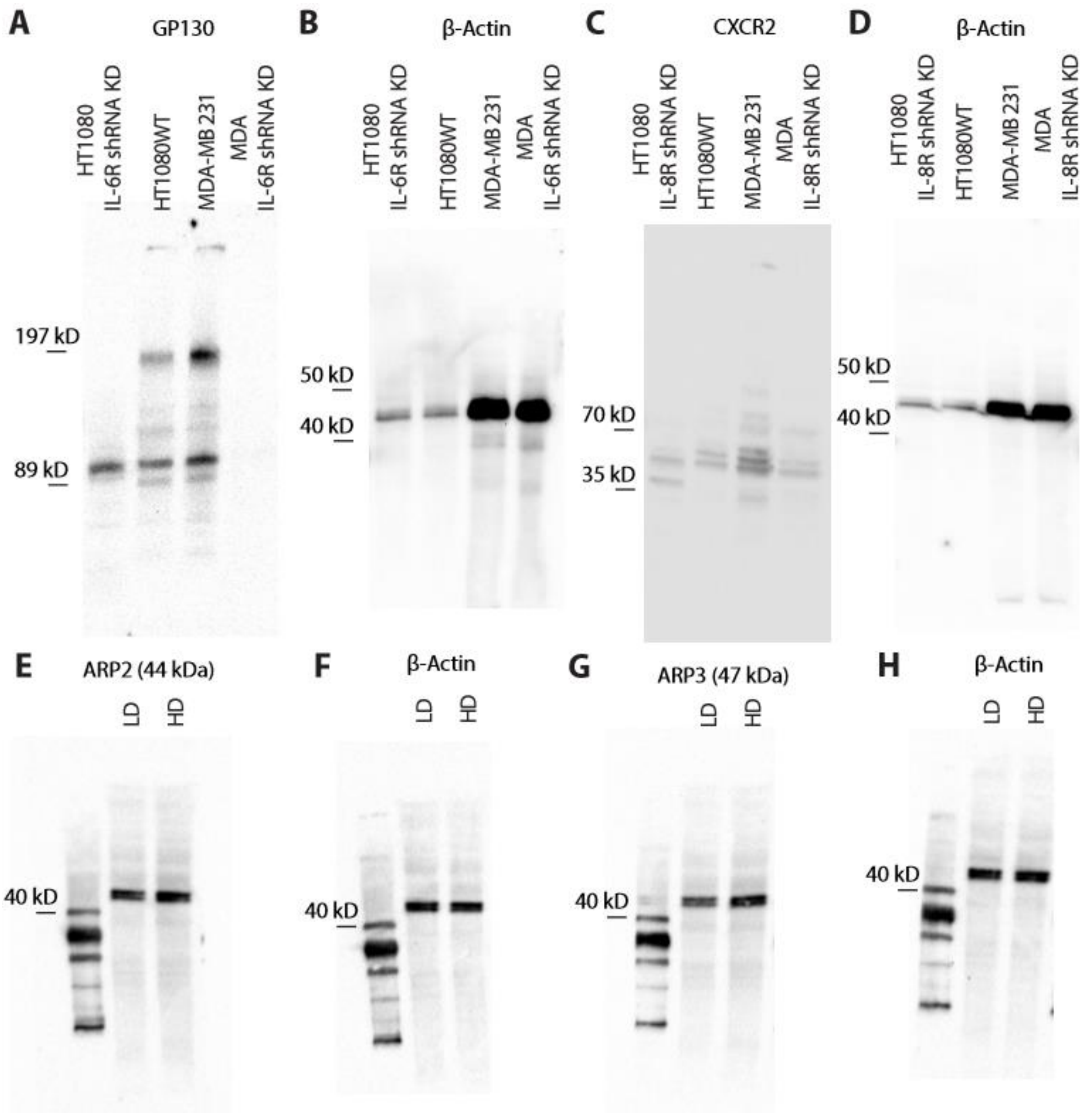

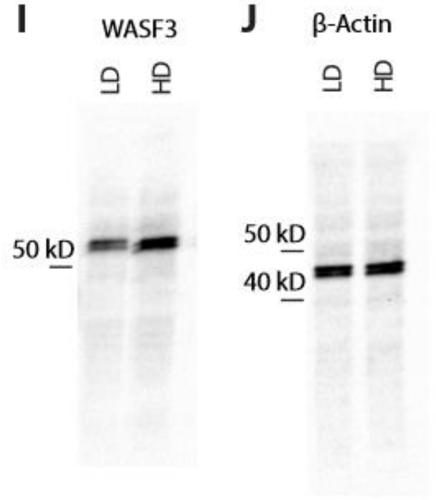

Supplementary Figure 6: Western blots A -D. Decreased expression of IL-6R and IL-8R in shRNA mediated knockdowns in HT1080 fibrosacroma and MDA-MB-231 carcinoma cells. **E-J.** ARP2, APR3, and WASF3 are upregulated under the high density condition in HT1080 fibrosarcoma cells.

IL-6 sh59205	CCGGCATCTCATTCTGCGCAGCTTTCTCGAGAAAGCTGCGCAGAATGAGATGTTTTTG;
IL-8 sh232053	CCGGTGCGCCAACACAGAAATTATTCTCGAGAATAATTTCTGTGTTGGCGCATTTTTG;
IL-6R sh289773	CCGGCCAGTCCAGATATTTCACATTCTCGAGAATGTGAAATATCTGGACTGG;
IL-8R sh378365	CCGGGAAGCGCTACTTGGTCAAATTCTCGAGAATTTGACCAAGTAGCGCTTCTT;
WASF3 sh344184	CCGGCGCTGCTATTCGAATGGGAATCTCGAGATTCCCATTCGAATAGCAGCGTTTTTG;
WASF3 sh381495	GTACCGGGCATCGGACGTTACGGATTACCTCGAGGTAATCCGTAACGTCCGATGCTTTTTTG;

Supplementary Table 1: Sequences for shRNA induced knockdown

HS-18S-FOR	GAGGATGAGGTGGAACGTGT
HS-18S-REV	AGAAGTGACGCAGCCCTCTA
HS-WASF3-FOR	AAGGGATTACCAGCGAACTTG
HS-WASF3-REV	CTTCAGCATGTTTGCTCAGACT
HS-ACTR2-FOR	CACCTGTGGGACTACACATTTG
HS-ACTR2-REV	TGGTTGGGTTCATAGGAGGTTC
HK2 FWD	CCAGTTCATTCACATCATCAG
HK2 RVS	CTTACACGAGGTCACATAGC

Supplementary Table 2: cDNA primers for QPCR
